# Application of Composite Soaking Solution in Fillet Storage and Caco-2 Cell Antioxidant Repair

**DOI:** 10.3390/foods14030442

**Published:** 2025-01-29

**Authors:** Qing Shao, Zhongqiang Wang, Shumin Yi

**Affiliations:** National & Local Joint Engineering Research Center of Storage, Processing and Safety Control Technology for Fresh Agricultural and Aquatic Products, College of Food Science and Engineering, Bohai University, Jinzhou 121013, China; shaoqing1939827103@163.com (Q.S.); wzq960908@163.com (Z.W.)

**Keywords:** compound soaking solution, sea bass, physicochemical quality, Caco-2 cell, oxidative stress injury

## Abstract

The inhibitory effect of compound soaking solution on the quality deterioration of fish fillets during storage and its repair effect on a cell oxidative damage model were investigated. Water holding capacity, cooking loss, thawing loss, thiobarbituric acid and sensory evaluation were used to verify that the composite soaking solution could improve the water loss and quality deterioration of fillets during frozen storage. At 180 d, water holding capacity was increased by 4.59% in the compound soaking solution group compared with the control. Cooking loss decreased by 6.47%, and thawing loss decreased by 13.06% (*p* < 0.05). The TBA value was reduced by 50%, and the degree of lipid oxidation was lower (*p* < 0.05). The results of the microstructure analysis showed that the tissue structure of fillets treated by the compound soaking solution was more orderly. The oxidative damage model of cells was achieved by soaking in treated fish fillet digestive juice, which inhibited the increase in reactive oxygen species content, maintained the integrity of the cell structure, and increased cell viability by 32.24% (*p* < 0.05). Compound soaking solution treatment could inhibit the quality deterioration of fish fillets during storage, and the digestive solution of fish fillets could improve the oxidative stress injury of Caco-2 cells induced by H_2_O_2_.

## 1. Introduction

Sea bass (*Lateolabrax japonicus*) belongs to Perciformes, which is widely distributed in the coastal areas of China, Japan, South Korea, and other countries. It is one of the most significant fish in terms of economic scale. The shelf life of sea bass can be extended by freezing. During the freezing storage of sea bass, protein denaturation, lipid oxidation, and ice crystal growth are significant contributors to the deterioration in quality of fish fillets caused by damage to their microstructure [1,2].

In order to prevent fish products from spoiling, protective freezing agents such as polysaccharides, phosphates, antifreeze proteins, and protein hydrolysate are typically added during processing [3]. Phosphate compounds (PCs) are widely used in the processing of aquatic products due to their functions such as water retention, texture improvement, antimicrobial properties, and nutritional enhancement [4,5]. As an antifreeze agent, PC mainly inhibits the quality deterioration of fish fillets by enhancing the protein network structure’s ability to retain moisture. The addition of PC can regulate pH, increase ionic strength, and inhibit the formation of disulfide bonds. Carbohydrates can increase the amount of water binding and improve the surface tension of water, avoiding protein denaturation under polar conditions [6]. Adding sugars during the freezing process can effectively reduce damage to the cell microstructure caused by ice crystal growth. Hydroxyl groups in trehalose (TR) form hydrogen bonds with polar groups of biomolecules, so that they can retain water and prevent water from freezing, preventing the aggregation and denaturation of proteins [7,8]. *Mirror carp*, *Litopenaeus vannamei*, and other aquatic products are protected by TR at low temperatures [4,9,10]. The addition of a single additive can only have an antifreezing and preservation effect from a certain perspective. However, in order to enhance the effectiveness, excessive use of a single additive may also lead to negative effects. Excessive addition of salt ions may cause changes in flavour, while excessive intake of salt may lead to cardiovascular diseases. Excessive addition of sugar will also bring about changes in flavour and an increase in energy. And antifreeze proteins and protein hydrolysates are limited in their use due to high costs and extraction methods. The combination of multiple antifreeze agents can reduce the amount of a single additive while also preventing quality deterioration in fish fillets during frozen storage from various angles. For example, a common antifreeze agent combination used in the freezing process of fish paste is a mixture of sucrose, sorbitol, and mixed phosphates [11]. During the freezing process, lipid oxidation products not only produce volatile compounds such as free fatty acids, aldehydes, ketones etc., but also cause protein denaturation. When we choose the widely used seaweed sugar for a soaking solution, not only does it have a low sugar content but it also possesses antioxidative properties. Sodium TR and tripolyphosphate can effectively inhibit the denaturation of myofibril-lar protein during freezing storage [12,13]. They possess antioxidant, anti-inflammatory, and lipid-lowering properties. The addition of phenolic extracts with other antioxidants may prevent the oxidation of proteins and the formation of free radicals [14,15,16]. TPs are composed of more than 30 phenolic substances, with the main components being catechins, flavonoids, and thearubigins [14]. In addition, the addition of tea polyphenols (TPs) further enhances the antioxidant effect of the soaking solution and can also inhibit microbial growth.

Lipid oxidation plays an important role in the quality of sea bass. Appropriate lipid oxidation enhances the flavour [17]. During storage, lipid oxidation may result in the accumulation of harmful substances, which may lead to increased consumption risk. By using a Caco-2 cell oxidative damage model, the antioxidant properties of CSS were further demonstrated, and the effects of CSS on health were evaluated. Caco-2 cells have similar functions as normal colon cells, so they can be used as a model for human intestinal physiology and pathology. Caco-2 cells represent a major cell model for exploring nutrient absorption in vivo [18,19].

This study aims to formulate a more effective composite soaking solution. By immersing sea bass fillets in the soaking treatment, it achieves inhibition of quality deterioration during freezing storage. In addition, this study further investigates the role of the compound soaking solution at the cellular level to determine if it has a sustained effect after fish fillets are digested.

## 2. Materials and Methods

### 2.1. Materials

Sea bass fillets were purchased from the Jinzhou Longyu Aquatic Products Distribution Office (Jinzhou, China). Phosphate compounds (sodium tripolyphosphate 50%, sodium pyrophosphate 20%, sodium hexametaphosphate 15%, disodium dihydrogen phosphate 5%, disodium hydrogen phosphate 5%, and trisodium phosphate 5%) were purchased from Henan Midaner Trading Co., Ltd. (Xinzheng, China). Tea polyphenol (containing 81.2% total TP, 72.6% total catechins, and >40% EGCG) was purchased from Zhejiang Yinuo Biotechnology Co., Ltd. (Quzhou, China). Trehalose was provided by Dezhou Huiyang Biotechnology Co., Ltd. (Dezhou, China). The Caco-2 cell line was derived from the Cell Resource Center, Institute of Basic Medicine, Chinese Academy of Medical Sciences (China). All other chemicals were at least of analytical grade.

### 2.2. Preparation of Fillets

Fillets were cut to form 17–18 g square shapes (2.5 × 2.5 × 1.5 cm; width × length × height). The fillets were immersed for 1 h in CSS. The solid–liquid ratio was 1:2. The whole process was carried out at 4 °C after removing excess water. The fish fillets were vacuum-packed and stored in the refrigerator at −20 °C.

### 2.3. Preparation of Simulated Digestive Juice In Vitro

Three steps (oral, gastric, and intestinal) were used for the in vitro gastrointestinal digestion [20]. The detailed preparation process of the simulated digestive fluid is described in Table A1 and Table A2. The in vitro digestion protocol steps were as follows: (i) oral phase, i.e., a 20 g homogenate sample was mixed with simulated saliva (SSF) (1:1) at 37 °C for 10 min.; (ii) stomach phase, i.e., simulated gastric fluid (SGF) was added to the oral phase sample to achieve a final volume ratio of 1:1. Subsequently, the mixture was rapidly adjusted to pH 3.0. The mixture was stirred at 37 °C for 2 h; and (iii) intestinal phase, i.e., the mixture after gastric digestion was adjusted to pH 7.0 and mixed with simulated intestinal fluid (SIF) at a ratio of 1:1. The mixture was stirred at 37 °C for 2 h to simulate intestinal conditions. At the end of gastric digestion, the samples were heated at 95 °C for 10 min. The samples were placed in an ice bath where enzymes were deactivated for 10 min and centrifuged (5424R, Eppendorf, Hamburg, Germany) for 10 min at 10,000 r min^−1^. Supernatants’ aliquots were collected and stored at −80 °C.

### 2.4. Cell Experimental Groups

CK-0 represents the medium containing cells and 250 μg mL^−1^ control group fish digestive juice. EG-0/EG-1/EG-2/EG-3/EG-4/EG-5 represent the medium containing cells and containing 0/50/125/250/500/1000 μg mL^−1^ compound soaking solution of fish fillet digestive juice.

### 2.5. Single-Factor Experiment

The cooking loss, water holding capacity, TBA, and sensory evaluation of the fillets were determined (the specific methods can be found in Section 2.7.2, Section 2.7.3, Section 2.7.6 and Section 2.7.7). The effects of different concentrations of TP, PC, and TR on fillet quality were investigated. The optimum concentrations of each of the components of the water retaining agent were determined through a single-factor experiment. The single-factor experimental groups are shown in Table A3.

### 2.6. Orthogonal Experiment

The TP, TR, and PC concentrations determined by the single-factor test were used in the orthogonal test for three kinds of food additives. A L_9_ (3^4^) orthogonal table (Table A3) was designed. At last, four indexes were evaluated: cooking loss, water holding capacity, TBA, and sensory evaluation to determine optimal CSS components.

### 2.7. Physicochemical Properties of Fillets After Soaking in CSS

#### 2.7.1. Thawing Loss

Frozen fish fillets after 30, 60, 90, 130, 150, and 180 days of storage at −20 °C in a refrigerator were thawed at 4 °C. Excessive water was removed using filter paper and the samples weighed before and after thawing [21]. Each group of samples was measured three times and the average value was taken. The calculation formula is as follows (1):

Thawing loss rate (%) = (m_1_ − m_2_)/m_1_ × 100 (1)

where m_1_ and m_2_ are the masses (g) of the sample before and after freeze–thawing, respectively.

#### 2.7.2. Cooking Loss

Cooking loss was investigated by weighing thawed fish fillets before and after steaming at 90 ± 2 °C for 20 min until the core temperature reached 70 °C. Each group of samples was measured three times and the average value was taken [22]. The calculation formula is as follows (2):

Cooking loss (%) = (M_1_ − M_2_)/M_1_ × 100 (2)

where M_1_ and M_2_ represent the weight of the fish fillet sample before and after cooking, respectively.

#### 2.7.3. Water Holding Capacity

The method of Shi et al., was slightly modified to determine the water holding capacity [23]. Approximately 1 g of fillets was weighed and marked as G_1_. The fillets were wrapped in filter paper and centrifuged. The mass after centrifugation was G_2_, with three parallels maintained in each group. The water holding capacity was calculated according to Formula (3):

Water holding capacity (%) = G_2_/G_1_ × 100 (3)

#### 2.7.4. Moisture Distribution and MRI Imaging Analysis

Fillets of 1 cm in diameter and 2 cm in height were loaded into nuclear magnetic tubes and then inserted into and measured in a low field NMR analyzer (NMI20, Shanghai Electronic Technology Co., Ltd., Shanghai, China) [24]. The parameter settings were as follows: SFI = 22 MHz, P90 = 14 μs, SW = 100 kHz, TR = 2000 ms, NS = 8, τ = 150 μs, Echocnt = 4000 and temperature condition: 32 °C. Magnetic resonance imaging experiments of fillets were also performed to obtain proton density-weighted images.

#### 2.7.5. pH

To measure the pH, 5 g of the fillets was mixed with 45 mL of distilled water and homogenized at 10 000 rpm for 2 min by using an FJ200 high-speed dispersing homogenizer (Shanghai Biaoben Mould Factory, Shanghai, China) [21]. Each group of samples was measured in parallel 3 times. The suspension was measured using an FE20 pH meter (Mettler Toledo, Shanghai, China).

#### 2.7.6. Thiobarbituric Acid

The TBA value of the fillets was determined by referring to the method of Siu et al. [2,25], where 3 g samples were homogenized (10,000 rpm, 4 °C, 1 min) with 15 mL of 10% trichloroacetic acid (TCA) solution. The reaction was carried out at 4 °C for 30 min, and the supernatant was filtered. The sample was mixed with TBA solution (0.02 M) in a 1:1 ratio and then heated in a water bath for 40 min. Then, the mixed sample was cooled to room temperature and we determined the absorbance of reactants at 532 nm and calculated the concentration of acetaldehyde according to the standard curve.

#### 2.7.7. Sensory Evaluation

The sensory evaluation group was composed of 10 experienced people in the laboratory, including 5 boys and 5 girls [15]. The fish fillets were scored according to Table A4, and the total sensory score was finally calculated (all assessors received written information about the study before the experiments and gave written voluntary consent to participate).

#### 2.7.8. HE Staining

Microstructure analysis sample preparation for microscopic analysis was conducted according to HE staining routine protocols [26], with a biological microscope (BHT-312, Olympus Co., Ltd., Tokyo, Japan) to observe extra-cellular space and fibre shrinking in tissue.

#### 2.7.9. Scanning Electron Microscopy

Scanning electron microscopy (S-4800, Hitachi Co., Ltd., Tokyo, Japan) was used to observe microstructure and morphology changes in the fillets [27]. The fillet was cut into a cuboid of 7 mm × 7 mm × 5 mm.

### 2.8. Determination of the Effect of the Digestive Fluid on the Caco-2 Injury Model In Vitro

#### 2.8.1. Cell Proliferation Activity

Of the complete medium, 100 μL was added to the blank and control groups, and 100 μL of the complete medium containing different concentrations of the fillet digestive fluid (0–1000 μg mL^−1^) was added to the experimental group. After 24, 48, and 72 h of cell culture, the 96-well plates were removed, respectively, and 10 μL CCK-8 solution was added to each well of the plate. The cells were shaken evenly using the cross-over method and incubated in the CO_2_ cell incubator in the dark for 2 h. The optical density (OD) value at 450 nm was measured using a microplate reader (Victor X3, Perkin Elmer, Inc., Shelton, CT, USA) [28]. The cell viability was determined using Formula (4).

Cell viability = As/Ac × 100(4)

where is the OD of cells cultured in medium which contains cells, culture medium, CCK-8, and the fillet digestive fluid, and Ac is the OD of cells cultured in medium which contains cells, culture medium, and CCK-8.

#### 2.8.2. Cytotoxicity Assay

The cells were cultured in an incubator for 24 h. The mixture was placed in a 96-well plate with CCK-8 solution in the dark. After 2 h of incubation in the dark, the OD values at 450 nm and 650 nm (a reference wavelength) were measured using the microplate reader [29]. Cytotoxicity was calculated using Formula (4).

#### 2.8.3. Establishment of the Oxidative Damage Model

The method of An Jun and Na J et al. was slightly modified to establish the oxidative damage model [30,31]. Cells were treated with different H_2_O_2_ concentrations (0, 250, 500, 750, 1000, 1500, 2000 μmol L^−1^). The treated cells were cultured in a cell incubator at 37 °C under 5% CO_2_ for 4 h to detect cell viability.

#### 2.8.4. Determination of the Mitochondrial Membrane Potential

Rhodamine 123 staining solution was added to the cells to prepare the final concentration of 10 μg mL^−1^. After the cells were incubated in the cell incubator for 30 min at 37 °C, they were washed twice with PBS and resuspended. The samples were placed in a quartz cuvette to determine fluorescence. The fluorescence intensity was measured using a fluorescence spectrophotometer (F-7000, Hitachi Co., Ltd., Tokyo, Japan). The excitation and emission wavelengths were 480–510 nm and 530 nm, respectively [32].

#### 2.8.5. Determination of Peroxy Radicals

Intracellular ROS production was detected using the ROS detection kit. The fluorescence intensity of the fluorescent product dichlorodihydrofluorescein diacetate (DCFH-DA) was proportional to the intracellular ROS level. Caco-2 cells were treated with the digestive fluid for 24 h, pretreated with 1000 μmol L^−1^ H_2_O_2_ for 4 h, and incubated with 10 μmol L^−1^ DCFH-DA at 37 °C for 30 min. The ROS levels were measured through laser confocal microscopy (Leica Stellaris 5, Leica Microsystems Co., Ltd., Wetzlar, Germany) and by using the fluorescence spectrophotometer [33].

#### 2.8.6. Observation of Cell Morphology

Caco-2 cells were cultured in six-well plates, and cell slides were added at the bottom of these plates in advance. After incubation, the slides were removed for hematoxylin–eosin (HE) staining and observed under an inverted microscope (AE2000, Motic China Group Co., Ltd., Xiamen, China).

### 2.9. Statistical Analysis

Each experiment was repeated three times, and the variance was examined. The results were expressed as mean ± standard deviation. According to the single-factor test results, the orthogonal test of L_9_ (3^4^) was carried out with Design-Expert software 13. Charts were prepared using Origin 2020 (OriginLab Corporation, Northampton, MA, USA). One-Way Analysis of Variance (ANOVA) and Tukey’s test were performed using SPSS (version 20.0, Chicago, NY, USA). The statistical significance was set at *p* < 0.05.

## 3. Results and Discussion

### 3.1. Single-Factor Experiment

#### 3.1.1. TP Concentration

**Figure 1:**
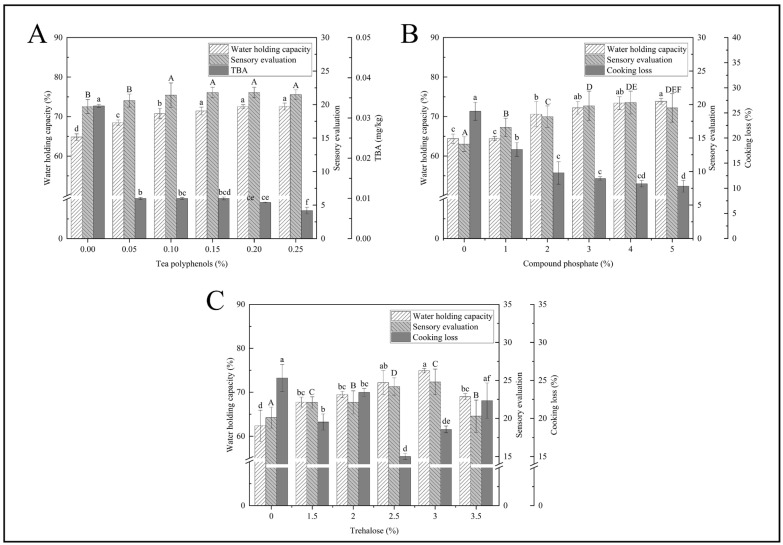
Water holding capacity, sensory evaluation, and TBA of fish fillets treated with different concentrations of TP (**A**); water holding capacity, sensory evaluation, and cooking loss of fillets treated with different concentrations of PC (**B**); water holding capacity, cooking loss, and sensory evaluation of fish fillets treated with different concentrations of TR (**C**). Note: different letters indicate significant differences (*p* < 0.05).

The water holding capacity of fillets increased with increasing concentrations of TP, which increased from 64.82% to 72.55% (*p* < 0.05) (Figure 1A). The TBA decreased significantly as TP concentration increased from 20.63 μg kg^−1^ to 9.77 μg kg^−1^, while TBA decreased slowly as TP concentration increased (*p* < 0.05). In fish fillets, protein oxidation results in damage to the amino acid structure, which leads to changes in protein conformation, partial water loss, and reduced water retention [34]. TP could inhibit protein oxidation and lipid oxidation and maintain tissue structure integrity [15,35]. There was a similar trend in the sensory characteristics and water retention capacity of the fillets, while TBA was opposite. The highest score of each sensory index was 21.8 at 0.15% concentration. Tea polyphenols inhibit lipid oxidation and microbial growth during the storage of fish fillets, reducing the generation of off flavours. The decrease in water holding capacity also affected the sensory evaluation of the testers on the fish fillets.

Based on the above analysis, the TP concentration was 0.1%, 0.15% and 0.2%, respectively.

#### 3.1.2. PC Concentration

Water retention capacity, sensory characteristics, and cooking loss were significantly affected by PC concentration (*p* < 0.05) (Figure 1B). With the increase in PC content, cooking losses decreased. The trend of the sensory evaluation shows an initial increase followed by a decrease. When the concentration was 4%, the testers gave the highest rating of 20.30, indicating that this level was the most acceptable (*p* < 0.05). The above results may be due to increasing the pH by adding phosphate, which results in a great distance from the isoelectric point of the protein, resulting in mutual repulsion between charges which provides more space, thus changing water retention capacity and cooking loss of the black bass fillets. This results in more water retention in the muscle, while higher ionic strength improves fish water retention [36]. Therefore, soaking in PC solution can effectively improve the moisture retention ability of fish fillets and enhance their sensory characteristics.

In order to extend the comparison between groups, 1%, 3%, and 5% PC solutions were chosen for subsequent orthogonal tests.

#### 3.1.3. TR Concentration

As the TR solution concentration increased, the water holding capacity and sensory characteristics of the fillets first increased and then decreased, and the cooking loss rate first decreased, then increased, then decreased, and again increased (Figure 1C). The cooking loss of the control group was obviously higher than the other group (*p* < 0.05). When the TR solution concentration was 2.5%, the cooking loss of the fish fillets decreased to 15.01%. At the 3% concentration, the water holding capacity increased to 74.92%, and the score of each sensory index was the highest, which was 24.8 (*p* < 0.05). This may be because TR has many hydroxyl groups that form hydrogen bonds with the polar groups of biomolecules, which helps aquatic products retain water and resist freezing [7,8]. When the concentration of trehalose exceeds 3%, the water holding capacity and sensory evaluation scores decrease slightly. An appropriate amount of seaweed sugar can effectively improve the water retention capacity, but excessive concentration may lead to changes in the structure of fish fillets, affecting their water retention capacity and decreasing sensory evaluation accordingly.

### 3.2. Orthogonal Test

As shown in Table 1, PC had the greatest effect on the water holding capacity and cooking loss of fresh fillets, followed by TR. Considering the influence of CSS on fillet quality, A_2_B_2_C_3_ (TR 3%, PC 3%, TP 0.2%) was determined. Verification experiments of the combination A_2_B_2_C_3_ were carried out. The sensory score increased to 22.50 points after being treated with soaking solution, and it decreased significantly to 12.15%. The water holding capacity increased to 80.89%, and TBA reduced to 2.23 μg kg^−1^ (*p* < 0.05).

### 3.3. Effect of CSS on the Quality of Fillets During Storage

#### 3.3.1. Water Holding Capacity, Cooking Loss, and Thawing Loss

**Figure 2:**
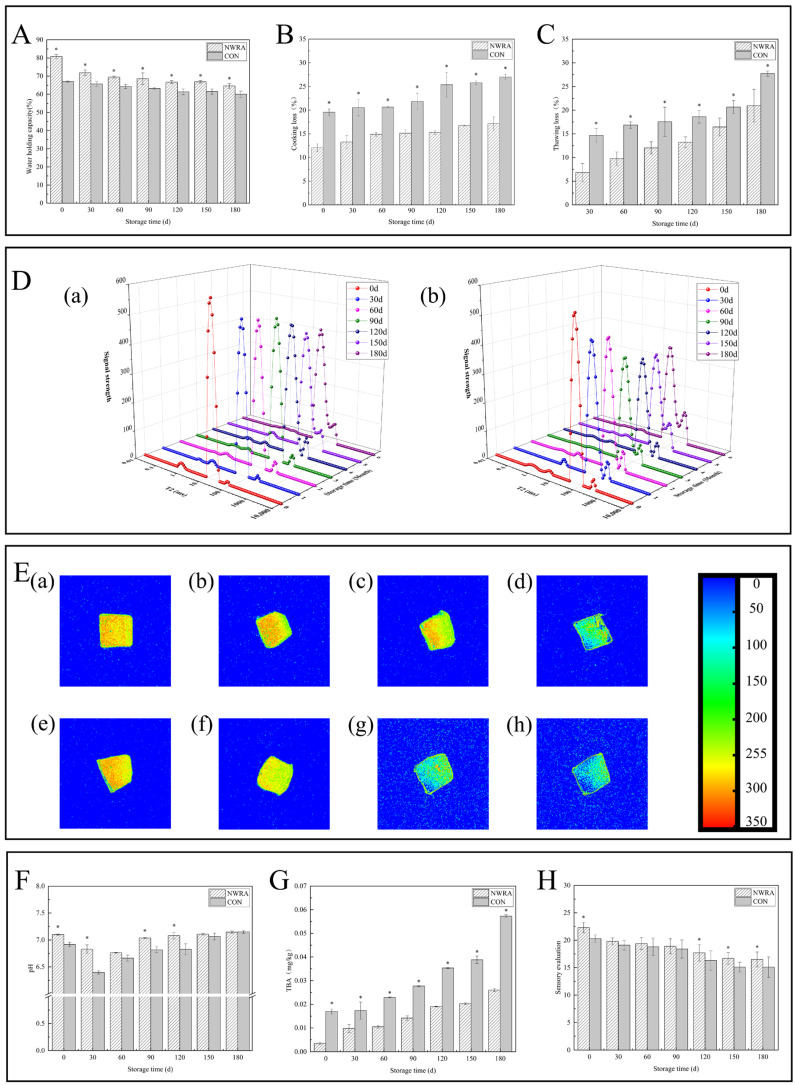
The effects of CSS on water holding capacity (**A**), cooking loss (**B**), thawing loss (**C**), water distribution (**D**), MRI imaging (**E**), pH (**F**), TBA (**G**), and sensory evaluation (**H**) of fillets. Among (**D**), (a) is the control group, (b) is the CSS group; among (**E**), (a–d) are the MRI images of CSS group at 0, 60, 120, and 180 days of frozen storage. (e–h) are the MRI images of the control group at 0, 60, 120, and 180 days of cryopreservation. Note: “*” indicate significant differences (*p* < 0.05).

Thawing loss and cooking loss decreased as the storage period prolonged, while the water holding capacity was opposite (Figure 2A–C). The reason for this is that during the freezing, different sizes of ice crystals form continuously in fish fillets, causing mechanical damage to cells [37]. During storage, the water holding capacity of samples treated with CSS was always higher than that in the control group. After 180 d, the water holding capacity increased by 4.59% and the cooking loss and thawing loss decreased by 9.85% and 6.76%, respectively (*p* < 0.05). The above changes are mainly due to the addition of the complex brining solution, which improves the ability of fish fillets to retain moisture under polar conditions. The TR in CSS can inhibit ice crystal growth and recrystallization during the freezing process, safeguarding the stability of the muscle structure and reducing thawing losses. During the association process, hydrogen bonds and hydrophobic interactions occur between the surface of ice crystals and trehalose, disrupting the ice layer, leading to solvation, and inhibiting the growth of ice crystals [9,38]. Furthermore, the salt ions in the soaking liquid increase the pH value of fish fillets, enhance electrostatic repulsion, and expand muscle fibres, thereby increasing water retention [39].

#### 3.3.2. Moisture Distribution and Migration

During storage, the proportion of immobilized water in the control group was significantly lower than that CSS (*p* < 0.05). In addition, the proportion of immobilized water decreased from 96.89% to 89.14% in the CSS group and decreased from 94.20% to 82.09% in the control group (Figure 2D). The third peak (pT_23_) obviously increased during storage (*p* < 0.05), which may be because of microbial growth and reproduction in the fish and damage to the structure of muscle fibres caused by enzymes and other substances produced during microbial metabolism in the fish. This led to the conversion of the water intercepted by muscle fibres into free water [40]. CSS can effectively suppress the above reaction and reduce the conversion of bound water to free water. A composite cryoprotectant composed of sucrose, sorbitol, and tripolyphosphate can reduce the loss of bound water during the freezing process by maintaining the structural integrity of proteins [41].

#### 3.3.3. Nuclear Magnetic Imaging Pseudo-Colour Image

During storage, the colour of the pseudo-colour images of the two fish fillet groups gradually changed from red to light red or yellow and finally to light blue or dark blue, thereby indicating that the water content of the samples was gradually reduced [42] (Figure 2E). This colour change was most obvious in the control group, which further verified that the proportion of non-flowing water in the CSS group was higher. At 0 d, the moisture distribution of the two groups of fish slices was similar. At 60 d–120 d, the fish slices treated with the soaking solution appeared red and yellow, indicating a high signal intensity and the highest water content. And the control group showed a rapid change in colour, from yellow to green, indicating significant loss of water during this process. And at 180 days, the blue part in the control group accounted for a large area, indicating serious loss of moisture. This indicates that the soaking treatment can better suppress the moisture loss of fish fillets, but it cannot completely prevent it.

#### 3.3.4. pH

The pH in the CSS group and control group decreased first and then increased (Figure 2F). The initial pH of the CSS group was 7.10, while that in the control group was 6.92. It may be due to the alkaline nature of CSS that the pH of fillets increased initially. During the whole storage period (except 150 days and 180 days), the pH in control group was obviously lower than CSS (*p* < 0.05). Initially, both groups experienced a decrease in pH, with the control group showing smaller changes. Soaking treatment can inhibit the degradation of glycogen and ATP inside fish meat, reducing the production of phosphoric acid and lactic acid. As storage time progresses, the degradation of proteins by microorganisms and endogenous proteases leads to an increase in pH due to the production of alkaline substances [43]. Compared to the control group, soaking treatment can inhibit the occurrence of the above reactions, so changes in the CSS group are smaller.

#### 3.3.5. TBA

The TBA increased first in both groups (Figure 2G). When stored for 180 days, the CSS group had a TBA of 0.03 mg kg^−1^ due to fatty oxidation. The TBA in the control group was 0.06 mg kg^−1^, twice as high as in the CSS group, and the fat oxidation degree was higher (*p* < 0.05). In the CSS group, there was a significant decrease in the lipid oxidation degree when compared to the control group. This is probably due to TP scavenging free radicals produced by lipid oxidation, thus delaying protein degradation, slowing down fillet quality degradation, and extending shelf life [35,44].

#### 3.3.6. Evaluation of Sensory Characteristics 

The sensory score decreased gradually during storage (Figure 2H). The scores of the CSS group reduced from 22.30 to 16.5, while those in control group reduced from 20.30 to 15.10. The sensory score of the control group was lower than CSS (*p* < 0.05). The flavour decreased slightly after 180 days, and there was a little fishy smell. The sensory characteristics of the fillets were within the acceptable range. It was found from the above analysis that CSS could effectively improve the sensory properties of bass fillets and inhibit quality degradation.

#### 3.3.7. Microstructure

**Figure 3:**
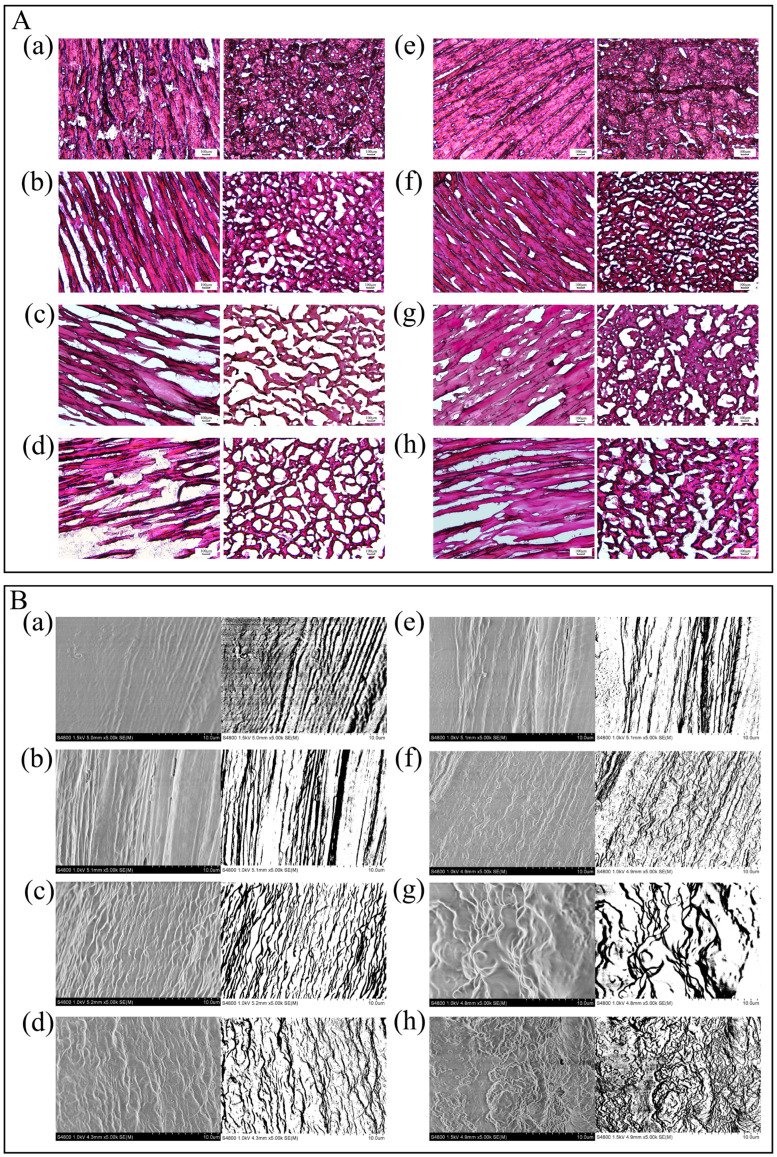
The effect of CSS treatment on the microstructure of fish fillets during storage was observed by inverted fluorescence microscope (**A**) and scanning electron microscope (**B**). Among them, (a–d) are the control groups at 0, 60, 120, and 180 days of storage; (e–h) are the composite soaking liquid groups at 0, 60, 120, and 180 days of storage, respectively.

At day 0, all group exhibited regular muscle fibres, full or minimally broken (Figure 3A,B). After 180 days, there were significant changes in the tissue structure in two groups. The control group showed gradual cracking and muscle fibres became loose. And there were many gaps in tissue structures. By contrast, in the CSS group, muscle fibre bundles were more closely arranged, with fewer fractures and fewer gaps. The results showed that CSS has a certain inhibitory effect on the deterioration of the fillet’s microstructure during fish storage.

In the freezing and thawing, ice crystals of various sizes formed continuously, and mechanical damage was caused by the ice crystals accumulated on the cells [37]. It is difficult for the injured myofibrils to absorb the dissolved water from the extracellular space, which leads to the partial transformation of the fixed water to free water. This is also the cause of the reduction in the water retention ability and the increase in the thawing loss. The muscle fibre structure of the control group began to be affected at 60 d, while the structure of the CSS group was orderly. After 120 d, the structure of the control group was damaged, and the muscle fibres were obviously deteriorated, which was also the cause of the decrease in the sensory score of this fish. However, the CSS group maintained a complete and orderly arrangement of the microstructure, which is closely associated with the excellent low temperature protective effect of TR. TR can inhibit the growth and recrystallization of the ice crystal, so that the structural stability of the muscle can be protected effectively [10]. The CSS group was intact, which somewhat inhibited the degradation of myofibrils and improved the quality of the fish. The improvement in the microstructure echoes that in the water holding capacity of fish fillets after the CSS treatment.

### 3.4. Effects of the Digestive Fluid of CSS-Treated Fillets on the Caco-2 Cell Injury Model In Vitro

#### 3.4.1. Detection of Cytotoxicity and Cell Proliferation

**Figure 4:**
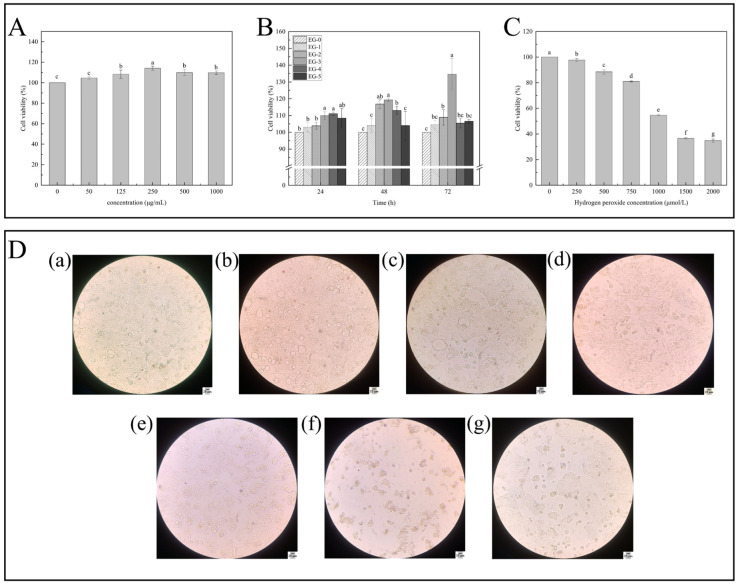
The effects of different concentrations of sea bass fillet digestive juice on Caco-2 cytotoxicity (**A**) and Caco-2 cell proliferation (**B**). Effects of different concentrations of H_2_O_2_ solution on the cytotoxicity of Caco-2 cells (**C**,**D**): (a–g) represent 0, 250, 500, 750, 1000, 1500, 2000 μmol L^−1^ of H_2_O_2_ solution on the morphology of Caco-2 cells. Note: different letters indicate significant differences between groups (*p* < 0.05).

The cell viability was more than 95%, and the viability of the experimental cells was higher than the control (*p* < 0.05) (Figure 4A). The concentration (0–1000 μg mL^−1^) did not damage the integrity of the cell during incubation, nor did it cause toxicity to Caco-2 cells. And the viability of cells was highest at 250 μg mL^−1^. The digestive fluid of the bass fillets containing phosphate affected Caco-2 cell activity.

In vitro, Caco-2 cell proliferation was influenced by concentrations of the digestive fluid and times of culture (Figure 4B). After 24 h, 48 h, and 72 h of digestion with 250 μg mL^−1^ concentration of digestive fluid, the viability of Caco-2 cells increased significantly. The viability of Caco-2 cells increased significantly at 72 h, reaching 134.58% (*p* < 0.05). Compared with the control group, 250 μg mL^−1^ digestive fluid had a significant effect on Caco-2 cell proliferation. Also, 0.23 mg mL^−1^ perch fillet digest added into cell culture medium could increase Caco-2 cell viability by 99.84% (*p* < 0.05) [29]. In the subsequent determination experiment, 250 μg mL^−1^ was chosen to be the concentration of the experimental group.

#### 3.4.2. Determination of Effects of H_2_O_2_ Concentration on the Caco-2 Cell Oxidative Damage Model

The H_2_O_2_ concentration of 0–2000 μmol L^−1^ significantly decreased the viability of cells (Figure 4C,D). As H_2_O_2_ concentration increased, cell viability appeared to decrease significantly (*p* < 0.05). The viability of Caco-2 cells was 54.64% when the H_2_O_2_ concentration was 1000 μmol L^−1^.

A model of oxidative damage was established based on cell survival rate [45]. When the H_2_O_2_ concentration was 1000 μmol L^−1^, Caco-2 cell viability was approximately 50%, which indicated that the oxidative damage model was successfully established.

#### 3.4.3. Antioxidant Activity

**Figure 5:**
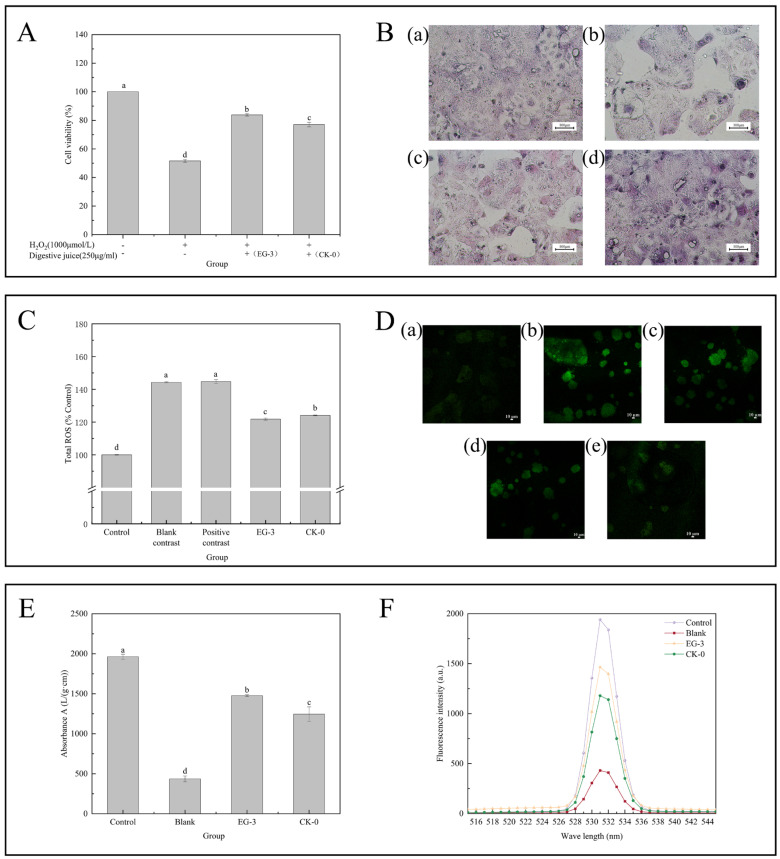
Protective effect of digestive fluid of sea bass fillets on oxidative damage of Caco-2 cells induced by H_2_O_2_ (**A**). The effects of sea bass fillet digestive solution on the morphology of Caco-2 cells injured by H_2_O_2_ (**B**): (a): only containing cells and culture medium; (b): cells + CK-0 group digestive fluid + H_2_O_2_; (c): cell + medium + H_2_O_2_; (d): cells + EG-3 group digestive fluid + H_2_O_2_. The effect of the digestive juice of sea bass fillets on the content of ROS in Caco-2 cells induced by H_2_O_2_: where (**C**) is the result of fluorescence spectrophotometer; (**D**) is the result of laser confocal measurement: (a) is the control group; (b) is the positive control group; (c) blank is the control group; (d) is the CK-0 group; and (e) is the EG-3 group. The effect of sea bass fillet digestive solution on the mitochondrial membrane potential of Caco-2 cells induced by H_2_O_2_ (**E**,**F**). Note: different letters indicates significant differences (*p* < 0.05).

The activity of Caco-2 cells decreased significantly after H_2_O_2_ treatment and without digestive fluid treatment (Figure 5A). The Caco-2 cells were significantly different in the intervention protection group treated with 250 μg mL^−1^ fillet digestive fluid. The Caco-2 activity of EG-3 and CK-0 cells reached (83.84 ± 0.82) % and (77.02 ± 1.59) % higher than those induced by the H_2_O_2_-induced oxidative damage group by 32.24% and 25.42%, respectively (*p* < 0.05). They did not reach the value in the control group. The addition of two groups of digestive juices, while not able to completely alleviate the oxidative stress damage caused by hydrogen peroxide, can effectively improve cell vitality. Among them, fish fillets treated with the soaking solution had a better digestive effect. This is because polyphenolic substances in the soaking solution can alleviate oxidative stress damage caused by hydrogen peroxide through various ways such as scavenging free radicals and promoting decomposition of hydrogen peroxide. When H_2_O_2_-induced oxidative damage cells were treated with bioaccessible fractions (BFs) from citrus pulp, BFs increased cell activity, but their activity was not as high as that of the controls [46].

#### 3.4.4. Cell Morphology Observation

The morphological changes in Caco-2 cells induced by H_2_O_2_ were observed by HE staining and optical microscope (Figure 5B). The cells of Caco-2 in blank group were better in terms of cell morphology and cell density (Figure 5B(a)). The oxidative damage cells of the EG-3 and CK-0 group were partially separated as compared with the group not treated with the digestive fluid. The number of oxidative damage cells with only medium was higher. It is suggested that digestive fluid can protect Caco-2 cells from oxidative damage and increase survival rate. Compared with CK-0, the digestive fluid from CSS-treated fillets could improve the morphology of Caco-2 cells damaged by H_2_O_2_-induced oxidative stress.

#### 3.4.5. Analysis of Changes in the ROS Content

Figure 5C shows that the intracellular ROS level in the H_2_O_2_-treated group increased significantly compared to the control group (*p* < 0.05). Normal growth was observed in the control group cells, and the ROS level was lowest. The highest level of ROS in the blank group and positive control group was 144.30% and 144.75%, respectively (*p* < 0.05). H_2_O_2_ induced cells to produce a large amount of ROS, while the ROS decreased significantly in the EG-3 group and CK-0 group (121.82% and 124.08%). In addition, compared with the CK-0 group, the ROS decreased significantly in the EG-3 group (*p* < 0.05). The digestive fluid retained the activities of succinate dehydrogenase and cytochrome-c reductase in mitochondria through alleviating damage caused by mitochondrial respiratory chain damage, reducing the increase in the intracellular level of ROS. In the CSS group, the digestion fluid (250 μg mL^−1^) significantly reduced ROS levels in Caco-2 cells. When mitochondrial ROS attack, proteins are oxidized so that carbonyl crosslinks or disulfide crosslinks can be formed. This changes the secondary or tertiary structure of the protein and induces protein unfolding. In addition, the oxidation of buried side chains may cause conformational changes [47]. Figure 5D shows that all the necrotic cells were stained successfully and exhibited green fluorescence. Moreover, a high concentration (250 μg mL^−1^) of digestive fluid could inhibit cell necrosis effectively. A reduction in fluorescent particles was observed under microscope.

ROS play an important role in cellular function. The ROS levels are positively correlated with the degree of damage to lipids, DNA, and proteins in cells. Therefore, reducing the level of ROS promotes cell proliferation [48,49]. The above results indicate that fillets’ digestive fluid can increase cell value-added through inhibiting H_2_O_2_-induced ROS production.

#### 3.4.6. Measurement of Mitochondrial Membrane Potential

Figure 5E,F show the effect of digestive fluid on the mitochondrial membrane potential of Caco-2 cells exhibiting oxidative damage. The mitochondrial membrane potential of CK-0 was lower than that of EG-3 (*p* < 0.05). Compared with the blank control group, there was a significant increase in the mitochondrial membrane potential in group EG-3 and CK-0 (*p* < 0.05). The mitochondrial membrane potential fluorescence intensity of the Caco-2 cells in the blank control group (436.07 ± 36.66) was 4.5 times lower than that in the control group (1963.33 ± 31.32) (*p* < 0.05). However, compared with the blank control group, the mitochondrial membrane potential dissipation was higher. It could not reach the baseline level observed in the control group. Mitochondrial dysfunction results in a loss of membrane potential, increased production of reactive oxygen species, and further apoptosis [50]. CSS-treated fillets exhibited significant resistance against mitochondrial damage induced by H_2_O_2_ in Caco-2 cells, which provided a theoretical basis for discovering the effect of CSS on the nutrient properties of aquatic products.

## 4. Conclusions

In this study, the optimal ratio of the composite soaking solution was determined to be 3.00% phosphate compounds, 3.00% trehalose, and 0.20% tea polyphenol. During storage, soaking can reduce the mechanical damage to the tissue structure of fish fillets caused by ice crystal growth, protect the integrity of the microstructure of fish fillets, and reduce the loss of water and nutrients in fish fillets. At the same time, the soaking solution can inhibit the degradation of unsaturated fatty acids and proteins during storage, inhibit the deterioration of fish fillet quality, and maintain good sensory evaluation. The oxidative damage model of Caco-2 cells was treated with fish fillet digestive juice. The results showed that the fish fillet digestive juice treated with soaking solution significantly improved the oxidative stress damage of Caco-2 cells induced by H₂O₂ and maintained the stability of mitochondrial membrane potential and the morphological integrity of Caco-2 cells. From the macroscopic aquatic product preservation scene to the microscopic cell level, the composite soaking solution demonstrates a high degree of consistency and continuity, revealing its potential in the field of food science and biomedicine, providing a reference for the study of oxidative damage-related diseases and offering new ideas for the prevention of intestinal diseases.

## Figures and Tables

**Table 1:** The results of orthogonal experiments.

Group	Trehalose (A) (%)	Phosphate Compound (B) (%)	Tea Polyphenols (C) (%)	Water Holding Capacity (%)	TBA (μg/kg)	Sensory Evaluation (Score)	Cooking Loss (%)
1	1	1	1	77.479	12.630	20.200	21.660
2	1	2	2	77.875	5.530	21.100	16.678
3	1	3	3	81.391	5.370	22.300	12.406
4	2	1	2	80.87	2.370	22.500	18.979
5	2	2	3	80.891	2.230	22.500	12.151
6	2	3	1	80.147	8.530	21.200	12.476
7	3	1	3	70.434	3.770	19.800	22.956
8	3	2	1	77.529	11.700	20.300	14.932
9	3	3	2	81.928	5.770	22.500	12.402
Water holding capacity							
K_1_	236.745	228.783	235.155				
K_2_	241.908	236.295	240.673				
K_3_	229.891	243.466	232.716				
R	12.017	14.683	7.957				
TBA							
K_1_	23.530	18.770	32.860				
K_2_	13.130	19.530	13.670				
K_3_	21.240	19.830	11.370				
R	10.400	1.060	21.490				
Sensory evaluation							
K_1_	63.600	62.500	61.700				
K_2_	66.200	63.900	66.100				
K_3_	62.600	66.000	64.600				
R	3.600	3.500	4.400				
Cooking loss							
K_1_	50.744	63.595	49.068				
K_2_	43.606	43.761	48.059				
K_3_	50.290	37.284	47.513				
R	7.138	26.311	1.555				

## Data Availability

The original contributions presented in the study are included in the article, further inquiries can be directed to the corresponding author.

## Appendix A

**Table A1:** Concentration of electrolytes in the electrolyte stock solution.

Electrolyte	Molar Concentration (mmol/L)		
	SSF	SGF	SIF
K^+^	18.80	7.80	7.60
H_2_PO_4_^−^	3.70	0.90	0.80
HCO_3_^−^, CO_3_^2−^	13.70	25.50	85.00
Cl^−^	19.50	70.20	55.50
Mg^2+^	0.15	0.10	0.33
NH_4_^+^	0.12	1.00	——
Na^+^	13.60	72.20	123.40
Ca^2+^	1.50	0.15	0.60

**Table A2:** Preparation method for digestive simulation fluid.

Digestive Fluid	Electrolyte Stock Solution (mL)	CaCl_2_ (mL)	pH	Deionized Water (mL)	Other Substances and Final Concentrations
SSF	16.00	0.10	7.00	3.90	α-amylase (0.81 mg)
SGF	36.40	0.02	1.60	3.58	Pepsin (10,000 U/mg) (16 mg)
SIF	74.00	0.16	7.00	5.84	Pancreatin (4000 U/g) (4 g); bile salts (0.65g)

**Table A3:** Single-factor experiments and orthogonal experiment.

	Levels	Factors		
		Tea Polyphenol (%)	Composite Phosphate (%)	Trehalose (%)
Single-factor experiment	1	0.05	1	1.5
	2	0.1	2	2
	3	0.15	3	2.5
	4	0.2	4	3
	5	0.25	5	3.5
Orthogonal experiment	1	2.50	0.10	1.00
	2	3.00	0.15	3.00
	3	3.50	0.20	5.00

**Table A4:** Sensory evaluation standard.

Item	1–3 Points	4–7 Points	8–10 Points
Hue	The surface is dull and the colour is dim.	The surface is slightly shiny and the colour is dark.	Lustrous surface, bright colour.
flavour	No fragrance, there is a smell. The fishy smell is heavier.	Lighter aroma. There is a small amount of astringency and fishy smell.	The fragrance is fresh. The comprehensive taste is good.
Texture	Low hardness, poor elasticity, and the concave part is slow to appear or does not disappear after pressing.	It has more hardness and elasticity, and the concave part gradually disappears after pressing.	Hardness is high, elastic, the depression part immediately disappears after pressing.
